# T-cell ligands modulate the cytolytic activity of the CD33/CD3 BiTE antibody construct, AMG 330

**DOI:** 10.1038/bcj.2015.68

**Published:** 2015-08-21

**Authors:** G S Laszlo, C J Gudgeon, K H Harrington, R B Walter

**Affiliations:** 1 Clinical Research Division, Fred Hutchinson Cancer Research Center, Seattle, WA, USA; 2 Department of Medicine, Division of Hematology, University of Washington, Seattle, WA, USA; 3 Department of Epidemiology, University of Washington, Seattle, WA, USA

## Abstract

Preclinical and emerging clinical studies demonstrate that bispecific T-cell engaging (BiTE) antibody constructs can potently lyse targeted tumor cells, but the determinants for their activity remain incompletely understood. Using human acute myeloid leukemia (AML) cell lines engineered to overexpress individual T-cell ligands, we found that expression of the inhibitory ligands, PD-L1 and PD-L2, reduced the cytolytic activity of the BiTE antibody construct targeting CD33, AMG 330; conversely, expression of the activating ligands, CD80 and CD86, augmented the cytotoxic activity of AMG 330. Consistent with these findings, treatment with an activating antibody directed at the co-stimulatory T-cell receptor, CD28, significantly increased AMG 330-induced cytotoxicity in human AML cell lines. Using specimens from 12 patients with newly diagnosed or relapsed/refractory AML, we found that activation of CD28 also increased the activity of AMG 330 in primary human AML cells (*P*=0.023). Together, our findings indicate that T-cell ligands and co-receptors modulate the anti-tumor activity of the CD33/CD3 BiTE antibody construct, AMG 330. These findings suggest that such ligands/co-receptors could serve as biomarkers of response and that co-treatment strategies with pharmacological modulators of T-cell receptor signaling could be utilized to further enhance the activity of this targeted therapeutic.

## Introduction

The use of antibodies that recognize both tumor cells and immune effector cells is a long-pursued strategy to improve antigen-specific immunotherapy of human cancers.^1, 2^ Although the clinical success of early molecules was limited, recent data obtained with bispecific T-cell engaging (BiTE) antibodies, which combine the minimal binding domains of the two different antibodies on one polypeptide chain, indicate that such small constructs can indeed have high anti-cancer activity. Binding the invariant epsilon subunit of CD3, they bring polyclonal CD3^+^ T-cells in close proximity of target tumor cells and force formation of an immunological lytic synapse that potently triggers lymphocyte activation and proliferation and, consequently, destruction of attached tumor cells through perforin/granzyme-mediated apoptosis.^2, 3, 4^

In clinical studies, a CD19/CD3 molecule built on this platform, blinatumomab, has shown very high efficacy in adults with relapsed or refractory CD19^+^ acute lymphoblastic leukemia, highlighting the therapeutic value of BiTE antibodies.^5, 6, 7^ A CD33/CD3 molecule, AMG 330, is currently in preclinical development for the treatment of acute myeloid leukemia (AML). In previous studies with AMG 330, we identified target antigen density, antibody dose and effector-to-target (E/T) cell ratio as critical determinants for the activity of the BiTE antibody construct in models of AML.^8^ So far, however, detailed mechanistic explorations of other factors that might contribute to clinical response or resistance to BiTE antibody constructs have not been conducted. Here, we used well-defined human CD33^+^ AML cell lines and genetically engineered sublines to test the impact of inhibitory (PD-L1 (B7-H1, CD274) and PD-L2 (B7-DC, CD273)) and activating (CD80 (B7-1) and CD86 (B7-2)) T-cell ligands on the cytolytic activity of AMG 330 *in vitro*, and then conducted proof-of-principle studies in specimens obtained from patients with AML.

## Materials and methods

### Generation of lentiviral vectors expressing T-cell ligands

Human cDNAs corresponding to CD80, CD86, PD-L1 or PD-L2 were generated via standard PCR cloning procedures (Supplementary Table 1) and verified by sequencing. CD80 (288 amino acids, corresponding to both CD80-001/ENST00000264246 and CD80-002/ENST00000478182 transcripts; no minor single-nucleotide polymorphisms (SNPs)), CD86 (329 amino acids, corresponding to CD86-001/ENST00000330540 with a minor SNP (185VtoI/rs2681417)) and PD-L2 (273 amino acids, corresponding to PDCD1LG2-001/ENST00000397747 with minor SNPs (F229S/rs7854303 and I241T/rs7854413)) cDNAs were cloned from THP-1 cells. PD-L1 (290 amino acids, corresponding to CD274-001/ ENST00000381577; no minor SNPs) cDNA was cloned from KG-1 cells.

### Parental and engineered human AML cell lines

The human CD33^+^ AML cell lines KG-1a, ML-1, TF-1 and HL-60 were maintained as previously described.^9, 10, 11^ Sublines of AML cell lines overexpressing T-cell ligands to various degrees were generated through transduction with a pRRLsin.cPPT.MSCV lentivirus^8, 11^ containing a human CD80-, CD86-, PD-L1- or PD-L2-IRES-Enhanced Green Fluorescent Protein cassette at a multiplicity of infection (MOI) of 0.25–25. Enhanced Green Fluorescent Protein-positive cells were isolated by flow cytometry and re-cultured for further analysis.

### Primary human AML cells

Frozen aliquots of Ficoll-isolated mononuclear cells from pretreatment (‘diagnostic') peripheral blood or bone marrow specimens from adult patients with AML were obtained from repositories at the Fred Hutchinson Cancer Research Center. We used the 2008 WHO criteria to define AML^12^ and the refined United Kingdom Medical Research Council criteria to assign cytogenetic risk.^13^ Patients provided written informed consent for the collection and use of their biospecimens for research purposes under protocols approved by the Fred Hutchinson Cancer Research Center Institutional Review Board. Clinical data were de-identified in compliance with Health Insurance Portability and Accountability Act regulations. Cells were cultured in Iscoves' Modified Dulbecco's medium (Life Technologies, Grand Island, NY, USA) supplemented with 20% fetal bovine serum (HyClone, Thermo Scientific, Logan, UT, USA) and 10 ng/ml each of interleukin-3, stem cell factor, granulocyte-colony-stimulating factor and granulocyte-macrophage colony-stimulating factor (all from Life Technologies).

### Healthy donor T cells

Unstimulated mononuclear cells were collected from healthy adult volunteers via leukapheresis under research protocols approved by the Western Institutional Review Board (Olympia, WA, USA). T-cells were enriched through magnetic cell sorting (Pan T-Cell Isolation Kit II; Miltenyi Biotec, Auburn, CA, USA), and then frozen in aliquots and stored in liquid nitrogen. Thawed cell aliquots were labeled with 3 μM CellVue Burgundy (eBioscience, San Diego, CA, USA) according to the manufacturer's instructions.^8^

### Quantification of drug-induced cytotoxicity

For experiments with AMG 330, cells were incubated at 37 °C (in 5% CO_2_ and air) in 96-well round bottom plates (BD Falcon) at 5–10 × 10^3^ cells/well in culture medium containing various concentrations of the BiTE antibody construct (kindly provided by Amgen, Amgen Research GmbH, Munich, Germany) as well as T-cells at different E/T cell ratios.^8^ In some experiments, blocking PD-L1 (clone 29E.2A3),^14^ PD-L2 (clone MIH18)^15^ or CD86 (clone IT2.2)^16^ antibodies, or an activating CD28 (clone CD28.2; all from BioLegend, San Diego, CA, USA) antibody were added at 1 μg/ml. After 48 h, cell numbers and drug-induced cytotoxicity, using 4′,6-diamidino-2-phenylindole (DAPI) to detect nonviable cells, were determined using a LSRII cytometer (BD Biosciences, San Jose, CA, USA) and analyzed with FlowJo (Tree Star, Ashland, OR, USA). Leukemic cells were identified by forward/side-scatter properties and negativity for CellVue Burgundy dye.^8^ For experiments with other cytotoxic agents, tumor cell lines were incubated in medium containing various concentrations of cytarabine or mitoxantrone (Sigma-Aldrich, St Louis, MO, USA) for 72 h, after which cell numbers and drug-induced cytotoxicity were quantified by flow cytometry.

### Immunophenotypic characterization of primary AML specimens

After thawing, cells were stained with directly labeled antibodies recognizing CD33 (clone P67.6; PE-conjugated), CD3 (clone SK7; PerCP-conjugated), CD45 (clone 2D1; FITC-conjugated), PD-L1 (clone MIH1, PE-conjugated), PD-L2 (clone MIH18, BV650-conjugated), CD86 (clone 2331, APC-conjugated), CD34 (clone 8G12; APC-conjugated; all from BD Biosciences) and CD80 (clone 2D10, PE-Cy7-conjugated; from BioLegend). To identify nonviable cells, samples were stained with DAPI. At least 10 000 events were acquired on an LSRII flow cytometer (BD Biosciences), and DAPI^-^ cells were analyzed using FlowJo.

### Statistical considerations

Linear median fluorescence intensity values were used to quantify expression levels of T-cell ligands. Drug-induced specific cytotoxicity is presented as: % cytotoxicity=100 × (1 – live target cells_treated_/live target cells_control_).^8^ AMG 330 cytotoxicity between control and CD28 antibody co-treated aliquots of primary human AML cells were compared using the Wilcoxon Signed-Rank test. Results from cytotoxicity assays are presented as mean values±standard error of the mean (s.e.m.) using Prism 6.0e (GraphPad, La Jolla, CA, USA).

## Results

To determine the impact of inhibitory and stimulatory T-cell ligands on the cytolytic activity of AMG 330, we selected several parental human CD33^+^ AML cell lines and engineered sublines that overexpress individual T-cell ligands as well-defined model systems. Based on our previous studies with AMG 330,^8^ cytotoxic effects of the BiTE antibody construct were assessed after 48 h in the presence of various antibody concentrations and healthy donor T-cells at different E/T cell ratios.

### Overexpression of inhibitory and stimulatory T-cell ligands modulates the cytolytic activity of AMG 330 in CD33^+^ AML cell lines

**Figure 1:**
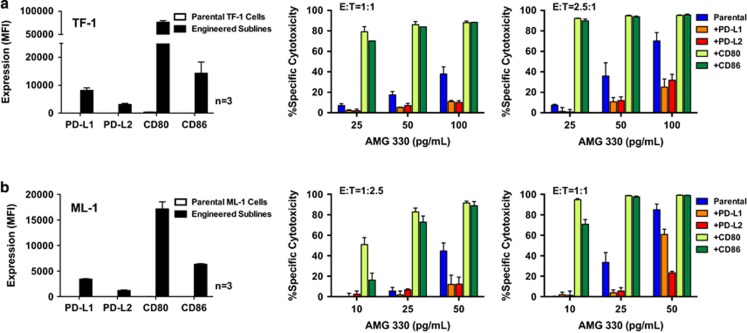
T-cell ligand overexpression modulates AMG 330-induced cytotoxicity in CD33^+^ human AML cell lines. (**a**) Parental TF-1 and (**b**) parental ML-1 cells were transduced with inhibitory (PD-L1, PD-L2) or stimulatory (CD80, CD86) T-cell ligands at a MOI of 25 to generate sublines that overexpressed single T-cell ligands, as indicated by arbitrary median fluorescence units. Parental cells and corresponding sublines were then incubated with increasing concentrations of AMG 330 and various E/T cell ratios using healthy donor T-cells. After 48 h, cell counts were determined and cytotoxicity was assessed with DAPI staining to quantify drug-specific cytotoxicity. Results (mean±s.e.m.) are shown as from three independent experiments for quantification of T-cell ligand expression and from three independent experiments performed in duplicate wells using a single healthy donor as source for exogenous T-cells for determination of specific cytotoxicity.

**Figure 2:**
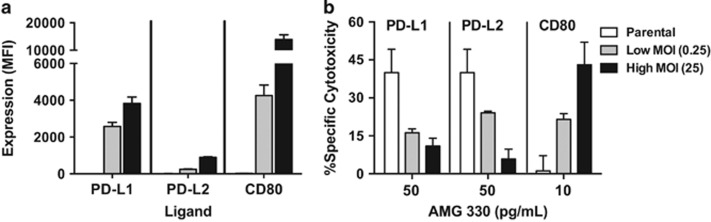
Effect of T-cell ligand expression levels on AMG 330-induced cytotoxicity. (**a**) Parental CD33^+^ ML-1 cells were transduced with PD-L1, PD-L2 or CD80 at a low MOI (0.25) or high MOI (25) to generate sublines that expressed individual T-cell ligands at different levels, as indicated by arbitrary median fluorescence units. (**b**) Parental cells and sublines were incubated with increasing concentrations of AMG 330 and healthy donor T-cells at an effector/target (E/T) cell ratio of 1:2.5. After 48 h, cell counts were determined and cytotoxicity was assessed with DAPI staining to quantify drug-specific cytotoxicity. Results (mean±s.e.m.) are shown from three independent experiments performed in duplicate wells.

In the presence of healthy donor T-cells, AMG 330 induced cytotoxicity in CD33^+^ TF-1 and ML-1 cells in a dose- and E/T cell ratio-dependent manner (Figure 1). With the exception of minimal expression of CD80 in TF-1 cells, these two human AML cell lines lacked expression of the inhibitory T-cell ligands, PD-L1, PD-L2 or the activating T-cell ligands, CD80, CD86. As depicted in Figure 1, lentiviral vector transfer of PD-L1, PD-L2, CD80 or CD86 into TF-1 or ML-1 cells at a MOI of 25 resulted in AML cell sublines that overexpressed single T-cell ligands. These sublines showed similar sensitivity to the cytotoxic effects of the two conventional AML therapeutics, cytarabine and mitoxantrone, as their parental cells (Supplementary Figure 1). However, sublines expressing the inhibitory ligand PD-L1 or PD-L2 were relatively resistant to AMG 330-induced cytotoxicity, as compared with corresponding parental cells. Conversely, sublines expressing one of the stimulatory ligands, CD80 or CD86, were significantly more sensitive to AMG 330 than parental cells (Figure 1). To test the dependency of this effect on the expression levels of the T-cell ligands, we generated a series of ML-1 sublines in which we expressed PD-L1, PD-L2 or CD80 at a low MOI (0.25) or high MOI (25). This approach resulted in sublines displaying various levels of individual T-cell ligands and demonstrated a quantitative relationship between the T-cell ligand expression and modulatory effect on AMG 330-induced cytotoxicity (Figure 2).

**Figure 3:**
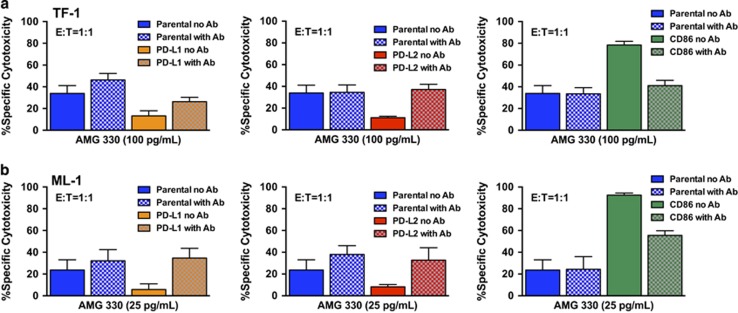
Antibodies block modulatory effect of T-cell ligands on AMG 330-induced cytotoxicity. Parental TF-1 (**a**) and ML-1 (**b**) cells and corresponding sublines transduced to overexpress individual T-cell ligands were incubated with AMG 330 and healthy donor T-cells in the presence or absence of corresponding blocking antibodies. After 48 h, cell counts were determined and cytotoxicity was assessed with DAPI staining to quantify drug-specific cytotoxicity. Results (mean±s.e.m.) are shown from four independent experiments performed in duplicate wells using a single healthy donor as source for exogenous T-cells.

These data indicated the overexpression of inhibitory or activating T-cell ligands could modulate the cytolytic activity of AMG 330. To study this effect in more detail and demonstrate its specificity, we incubated TF-1 and ML-1 sublines overexpressing either PD-L1, PD-L2 or CD86 with corresponding antibodies blocking the interaction of these T-cell ligands with their receptors. As shown in Figure 3, co-treatment with anti-PD-L1, anti-PD-L2 or anti-CD86 antibodies abrogated the effect of these ligands on AMG 330-induced cytotoxicity, further supporting the notion that the redirection of T cells toward AML cells is influenced by the expression of T-cell ligands.

### Pharmacological activation of the co-stimulatory T-cell receptor, CD28, enhances the cytolytic activity of AMG 330 in AML cell lines

**Figure 4:**
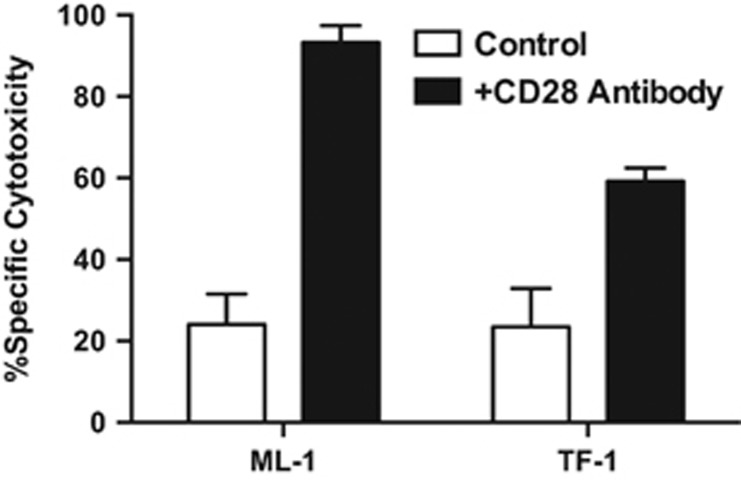
Activation of CD28 enhances AMG 330-induced cytotoxicity in human AML cell lines. Parental CD33^+^ AML cell lines (ML-1 and TF-1) were incubated in the presence of AMG 330 (50 pg/ml) and healthy donor T-cells at an E/T cell ratio of 1:1 in the presence or absence of an activating CD28 antibody clone (CD28.2). After 48 h, cell counts were determined and cytotoxicity was assessed with DAPI staining to quantify drug-specific cytotoxicity. Results (mean±s.e.m.) are shown as from three independent experiments performed in duplicate wells using a single healthy donor as source for exogenous T-cells for determination of specific cytotoxicity.

These initial findings suggested the possibility that pharmacological activation of co-stimulatory T-cell receptors (for example, CD28, which binds to CD80 and CD86)^17^ could augment the anti-tumor activity of AMG 330 and, consequently, could provide a rational means of increasing the efficacy of these agents. To test this concept, we determined the cytolytic activity of AMG 330 on AML cells in the presence or absence of an activating CD28 antibody. Indeed, as shown in Figure 4, leukemic cell lines co-treated with this antibody were significantly more susceptible to the BiTE antibody construct.

### Pharmacological activation of CD28 enhances the cytolytic activity of AMG 330 in primary AML specimens

Having established that activation of CD28 enhances the activity of AMG 330 in leukemic cell lines, we aimed to assess whether activation of CD28 also impacted the efficacy of BiTE antibody constructs against primary patient leukemia cells. To this end, we obtained specimens from 17 patients with newly diagnosed (*n*=9) or relapsed/refractory (*n*=8) AML for our studies. Upon thaw, 14 had >40% AML blasts, as determined by flow cytometry based on CD45/side-scatter properties. Twelve of these fourteen specimens had >50% viable cells upon thaw and >30% viable cells after 48 h in cytokine-containing liquid culture and were included in our analyses (Table 1). The median age of these patients was 68.0 (range: 58.1–83.1) years; cytogenetic disease risk was intermediate in 9, adverse in 1 and unknown in 2. The median percentage of myeloid blasts and CD3^+^ T-cells in the studied specimens was 88.9% (range: 49.3–97.1%) and 2.4% (range: 0–12.5%), respectively, and the median sample viability after 48 h in culture was 82.3% (range: 41.2–95.8%). As summarized in Table 1, there was generally very low to absent expression of CD80, CD86, PD-L1 and PD-L2 on AML blasts upon thawing. Consistent with this, AMG 330-induced cytotoxicity was not significantly affected by co-treatment with antibodies blocking CD80 and/or CD86 or antibodies blocking PD-L1 and/or PD-L2 (data not shown). On the other hand, co-treatment with an activating CD28 antibody statistically significantly increased AMG 330-induced cytotoxicity overall (*P*=0.023), with a >10% absolute increase in specific cytotoxicity in 4/12 specimens, and a >50% relative increase in specific cytotoxicity in 7/12 specimens, respectively (Table 1).

## Discussion

Emerging data with blinatumomab indicate that BiTE antibody constructs can be highly effective in some patients with chemotherapy-refractory acute lymphoblastic leukemia. Still, in a significant number of individuals, the BiTE antibody construct has insufficient activity despite expression of CD19 on leukemic cells.^5, 6, 7^ Results from a large multicenter phase 2 trial of blinatumomab monotherapy suggest that the tumor burden present at the time of therapy may be one critical determinant for the clinical activity of BiTE antibody constructs.^7^ However, other determinants of response have yet to be identified. Undoubtedly, detailed knowledge of such factors would be instrumental not only for the identification of suitable biomarkers for risk-stratified treatment decision-making and response prediction but also for the development of rational combination therapies aimed to overcome relative resistance to BiTE antibody constructs and augment their clinical activity.

As a first attempt at understanding cancer cell-associated regulators of BiTE antibody construct-mediated cytotoxicity, we performed well defined and controlled cell line experiments to investigate how AMG 330-induced tumor cell destruction is impacted by several B7 family members, namely CD80, CD86, PD-L1 and PD-L2, and signaling through the activating co-receptor, CD28; we were interested in the latter as it is well recognized that interactions of CD80 and CD86 with CD28 on T cells generate signals that are important to augment T-cell immune responses.^17, 18^ For other formats of bispecific antibodies (for example, hybrid antibodies generated by quadrome technology or covalently coupled F(ab')2 fragments), multiple studies have demonstrated that T-cells are insufficiently activated without co-stimulatory signal via, for example, the concomitant use of bispecific or monospecific antibody directed at co-stimulatory receptors such as CD28.^19, 20, 21, 22^ For BiTE antibody constructs, it is well established that potent target cell cytolysis can occur in the absence of pharmacologic stimulation of T-cell co-receptors.^8, 23, 24, 25, 26, 27^ This observation has suggested the possibility that the cytolytic property of BiTE antibody constructs are entirely independent of T-cell co-receptor activity, perhaps because of the tightness of the immunological lytic synapse that is forced between the target tumor cell and T-cell by the BiTE antibody construct. As demonstrated in this study, in which CD33^+^ AML cell lines lacking detectable expression of CD80 or CD86 were efficiently killed by AMG 330, *in vitro* cytotoxicity induced by BiTE antibody constructs is indeed independent of CD80 or CD86-induced T-cell activation. Nonetheless, as the major finding of our current studies, expression of CD80 or CD86, or direct stimulation of CD28 with an activating antibody, does provide a signal that leads to drastically increased activity of BiTE antibody constructs, as shown for AMG 330. These data are consistent with limited studies with tetravalent tandem diabodies, in which cancer cell expression of CD80 or CD86 or concomitant treatment CD28 antibodies led to significantly increased activity of the bispecific antibody.^28, 29^ Conversely, cancer cell expression of PD-L1 or PD-L2, which are thought to act exclusively as negative regulatory ligands,^18^ noticeably decreased the cytotoxic activity of AMG 330. In AML cells engineered to overexpress various amounts of individual T-cell ligands, we found this effect to be proportional to the amount of ligand expressed and not to be an on/off phenomenon.

As strength of our study, we largely used engineered AML cell lines that enabled us to manipulate the expression of individual factors of interest and conduct well-controlled mechanistic experiments. As a limitation, our experiments depended on lentivirus-mediated overexpression of T-cell co-receptors and involved allogeneic T cells. Nevertheless, our findings that T-cell ligands can modulate the cytolytic activity of AMG 330 have at least two implications. First, our data suggest that expression profiles of one or more of these ligands could serve as biomarker of clinical response. This hypothesis could be prospectively or retrospectively tested in specimens collected from patients treated with a BiTE antibody construct. Our findings would support the conduct of such correlative studies, which could then, in turn, provide validation for our observations made in engineered cell lines. These studies would not only need to validate our findings in the clinic but also identify the optimal time point for the determination of the T-cell ligand profile. As the expression of T-cell ligands is dynamic and can change fluidly as a response to external factors, including cytokines and prolonged exposure to BiTE antibody constructs,^30^ it will be important to assess the value of T-cell ligand profiles as response biomarker not only on samples obtained at baseline before initiation of BiTE antibody construct therapy—unquestionably the most convenient and helpful time point for risk-stratified treatment decision-making—but perhaps also on cancer cells obtained during BiTE antibody construct treatment.

As a second implication, our data suggest that manipulation of T-cell co-receptor signaling could serve as a viable strategy to improve the activity of AMG 330 and, by extrapolation, other BiTE antibody constructs, and overcome relative resistance in patients who otherwise would experience insufficient drug efficacy. This notion is supported by our findings with antibodies blocking PD-L1 or PD-2 (in engineered acute leukemia cell lines expressing these ligands) as well as our findings with an activating CD28 antibody in human AML cell lines and, more importantly, several primary specimens from patients with AML. Interestingly, although we used healthy donor T-cells from one individual in our comparative analyses, the effects of the CD28 antibody varied considerably across the AML cell lines and the 12 primary AML specimens tested, suggesting that one or more currently unidentified cancer cell-related factor(s) can further modulate the interaction with the CD28-activated T-cells; this modulation will need further investigation in future studies. Of note, our ability to test the potential of a combination approach between AMG 330 and a pharmacological agent that modulates T-cell co-receptor signaling was somewhat limited in our experimental system as neither our AML cell lines nor the primary AML cells selected for our studies constitutively expressed T-cell ligands at significant levels. However, increasing evidence indicates that such ligands can be displayed on malignant myeloid or lymphoid cells in patients with active leukemia before treatment initiation and/or be induced by various stimuli such as cytokines, histone deacetylase inhibitors, DNA methyltransferase inhibitors and conventional chemotherapeutics.^31, 32, 33, 34, 35, 36, 37, 38, 39^ Consistent with this, we observed significantly increased expression of T-cell ligands after 24–48 h culture of several of the primary AML cells in the presence of interferon-γ (Supplementary Figure 2). As several checkpoint inhibitors (for example, antibodies blocking CTLA-4 or PD-1) are currently undergoing testing as a means to restore anti-tumor immune responses in human trials,^18, 40^ it is conceivable that the clinical potential of such combination strategies could be exploited in the near future.

In summary, our data show that expression of T-cell ligands and signaling through T-cell co-stimulatory or co-inhibitory receptors modulate the anti-tumor activity of AMG 330. These findings support our conclusion that expression of such ligands/co-receptors should be explored in clinical studies as potential biomarkers of response. Moreover, our studies provide the rational for the use of pharmacological approaches to modulate T-cell co-receptor signaling as a means to augment the activity of AMG 330 and, likely, other BiTE antibody constructs.

## Figures and Tables

**Table 1:** Characteristics of primary AML specimens and effect of activating CD28 antibody on AMG 330-induced cytotoxicity

*Sample*	*Age (years)*	*Cytogenetic risk*	*Baseline phenotyping*							*Specific cytotoxicity*	
			*AML blasts (%)*	*CD33 (MFI)*	*PD-L1 (MFI)*	*PD-L2 (MFI)*	*CD80 (MFI)*	*CD86 (MFI)*	*CD3^+^ cells (%)*	*Control*	*+CD28 antibody*
1	63.0	Intermediate	94.0	1059	21	0	19	6	1.2	10.3	18.8
2	65.7	Intermediate	91.0	408	25	3	9	51	1.3	24.1	38.7
3	58.1	Adverse	97.1	4842	33	5	34	244	0.0	68.0	70.5
4	67.1	Intermediate	79.1	3138	19	-5	50	-1	7.5	5.1	15.8
5	71.5	Intermediate	89.3	2159	25	4	74	66	3.7	16.5	28.7
6	65.3	Intermediate	88.5	171	35	4	68	15	0.9	3.9	9.8
7	58.5	Not available	91.1	431	36	-2	81	50	1.0	0	0
8	78.0	Intermediate	95.1	54	9	0	-6	12	1.0	10.9	12.5
9	76.4	Intermediate	82.0	363	29	3	196	60	3.6	20.0	18.7
10	68.8	Intermediate	49.3	3316	55	7	114	213	12.5	3.8	7.0
11	73.4	Intermediate	63.2	24	26	-6	7	1	10.5	16.7	8.2
12	83.1	Not available	50.7	1171	26	-33	60	39	6.0	16.7	43.9

Drug-specific cytotoxicity is shown for AMG 330 at a concentration of 50 pg/ml in the presence of healthy donor T-cells at an E/T cell ratio of 1:1.

Abbreviations: AML, acute myeloid leukemia; MFI, median fluorescence intensity.

## Notes

RBW has received research funding from Amgen, Inc., Amphivena Therapeutics, Inc. and Seattle Genetics, Inc., and has been a consultant for Amphivena Therapeutics, Inc., AstraZeneca, Inc., Covagen AG and Pfizer, Inc. The remaining authors declare no conflict of interest.
