# Supplementary material for: T-cell ligands modulate the cytolytic activity of the CD33/CD3 BiTE antibody construct, AMG 330
Source: Blood Cancer J. 2015 Aug 21;5(8):e340–. doi: 10.1038/bcj.2015.68 (PMC4558592; doi:10.1038/bcj.2015.68)
Supplement: Supplementary Information [file bcj201568x1.docx]

**SUPPLEMENTAL TABLE 1. List of Cloning Primers.**

| **T-cell Ligand** | **Cloning Primers** |
| --- | --- |
| **CD80** | For: ATGGGCCACACACGGAGG  Rev: TTATACAGGGCGTACACTTTCCCTTC |
| **CD86** | For: ATGGATCCCCAGTGCACTATG  Rev: TTAAAAACATGTATCACTTTTGTCGC |
| **PD-L1** | For: ATGAGGATATTTGCTGTC  Rev: TTACGTCTCCTCCAAATG |
| **PD-L2** | For: ATGATCTTCCTCCTGCTAATG  Rev: TCAGATAGCACTGTTCACTTC |

**SUPPLEMENTAL FIGURE 1**

**Effect of inhibitory and stimulatory T-cell ligands on cytarabine- and mitoxantrone-induced cytotoxicity in human CD33+ AML cell lines.** Parental TF-1 **(A)** and ML-1 **(B)** cells and corresponding sublines overexpressing individual T-cell ligands (PD-L1, PD-L2, CD80, or CD86) were incubated with increasing concentrations of cytarabine or mitoxantrone as indicated. After 72 hours, cell counts were determined and cytotoxicity was assessed with DAPI staining to quantify drug-specific cytotoxicity. Results are shown as mean±SEM from 3 independent experiments performed in duplicate wells.

**SUPPLEMENTAL FIGURE 2**

**Effect of IFNγ on T-cell ligand expression on primary human AML cells.** Parallel aliquots of 9 primary human AML specimens (sample numbers corresponding to specimens described in Table 1) were incubated in regular culture medium supplemented with either IFNγ (10 or 100 ng/mL) or regular culture medium only. After 48 hours (samples #1-3) or 24 hours (other samples), expression of **(A)** PD-L1, **(B)** PD-L2, **(C)** CD80, and **(D)** CD86 was quantified by flow cytometry. Results are shown as arbitrary median fluorescence units.
